# Game-Based Assessment of Cognitive Abilities and Personality Characteristics for Surgical Resident Selection: A Preliminary Validation Study

**DOI:** 10.2196/72264

**Published:** 2025-08-15

**Authors:** Noa Gazit, Gilad Ben-Gal, Ron Eliashar

**Affiliations:** 1 Department of Prosthodontics, Faculty of Dental Medicine, Hebrew University of Jerusalem, Hadassah Medical Center, Kalman Ya'akov Man 1, Jerusalem, Israel, 972 547567448; 2 Department of Otolaryngology/HNS, Faculty of Medicine, Hebrew University of Jerusalem, Hadassah Medical Center, Jerusalem, Israel

**Keywords:** resident selection, assessment, surgical training, cognitive abilities, personality characteristics, gamification, game-based assessment

## Abstract

**Background:**

Assessment of nontechnical attributes is important in selecting candidates for surgical training. Currently, these assessments are typically made based on ineffective methods, which have been shown to be poorly correlated with later performance.

**Objective:**

The study aimed to examine preliminary evidence regarding the use of game-based assessment (GBA) for assessing cognitive abilities and personality characteristics in candidates for surgical residencies.

**Methods:**

The study had 2 phases. In the first phase, a gamified test was developed to assess competencies relevant for surgical residents. Three games were chosen, assessing 14 competencies: planning, problem-solving, ingenuity, goal orientation, self-reflection, endurance, analytical thinking, learning ability, flexibility, concentration, conformity, multitasking, working memory, and precision. In the second phase, we collected data from 152 medical interns and 30 expert surgeons to evaluate the test’s feasibility, acceptability, and validity for candidate selection.

**Results:**

Feedback from the interns and surgeons supported the relevance of the test for selection of surgical residents. In addition, analyses of the interns’ performance data supported the appropriateness of the score calculation process and the internal structure of the test. Based on this data, the test showed good psychometric properties, including reliability (α=0.76) and discrimination (mean discrimination 0.39, SD 0.18). Correlations between test scores and background variables indicated significant correlations with gender, video game experience, and technical aptitude test scores (all* P*<.001).

**Conclusions:**

This study presents an innovative GBA testing cognitive abilities and personality characteristics. Preliminary evidence supports the validity, feasibility, and acceptability of the test for the selection of surgical residents. However, evidence for test-criterion relationships, particularly the GBA’s ability to predict future surgical performance, remains to be established. Future longitudinal studies are necessary to confirm its utility as a selection tool.

## Introduction

Selection of surgical training residents is an essential process aimed at ensuring that only the most capable candidates are chosen to undergo the rigorous training required to become qualified surgeons. Alongside technical skills, there is broad consensus that it is also crucial to assess nontechnical attributes, including cognitive abilities (eg, deductive reasoning, learning ability, and concentration) and personality characteristics (eg, decision-making, stress tolerance, and communication skills), in potential surgical residents [1-7]. Indeed, some even consider nontechnical attributes to be more relevant for selecting surgical trainees than technical aptitude [7-9]. In a recent study [7], 19 nontechnical competencies were identified as relevant to surgeons in the 21st century (6 cognitive abilities and 13 personality characteristics).

Traditionally, surgical training programs have assessed nontechnical attributes almost exclusively through proxies such as academic achievement, curricula vitae, letters of recommendation, and unstructured interviews [10,11]. However, studies suggest that these methods are poorly correlated with later performance during residency [11-16]. In light of such findings, some studies have examined the use of self-report measures as a potential alternative. For example, studies have explored the potential of self-report questionnaires for assessing personality, emotional intelligence, and grit. But there is as yet no consistent evidence that these methods improve the selection of surgical residents [5,17]; and these tools are subject to all the potential problems and biases of self-reports, from poor introspective ability to outright dishonesty [18,19]. Hence, better ways of assessing surgical residency candidates are needed.

One promising new approach is to analyze behavior itself using simulated tasks, where examinees are exposed to controlled situations designed to elicit behaviors relevant to the assessment of specific competencies. This method is expected to have higher predictive value than either traditional methods or self-reports.

A simulation test can be conducted in the real world by evaluators or actors, or on a computer using emerging technologies such as virtual reality and gamification. Gamification refers to the incorporation of game elements into nongaming activities, and its application to personnel selection has led to the development of game-based assessments (GBAs). GBAs use gameplay behaviors to assess job-related skills, abilities, and characteristics, and they have many advantages over traditional assessments and noncomputerized simulation tests for predicting job performance [20-23]. First, GBAs promote a more positive assessment experience that reduces examinees’ stress levels and increases their engagement and motivation. Second, GBAs are based on an automated scoring system, which eliminates the bias often associated with human assessments. Finally, GBAs can collect rich high-resolution spatiotemporal data capturing examinees’ behavior throughout the test, allowing the entire solving process to be examined rather than just the final result or answer. These advantages may lead to a more reliable and valid assessment of examinees’ skills and abilities.

As GBAs are still relatively new, only a limited number of studies have examined their use in hiring and recruitment [24-26], and to the best of our knowledge, no study has evaluated GBAs as a tool for selecting medical residents. The current study examines the use of GBA for assessing cognitive abilities and personality characteristics identified as relevant for surgical residents in an initial phase of job analysis [7]. This study is the first in a planned series of studies aimed at establishing the validity of the GBA. Here, we present preliminary evidence of its feasibility, acceptability, and validity in the context of surgical resident selection, based on feedback and behavioral data from potential candidates and expert surgeons. Further research linking the GBA scores to future surgical performance will be necessary to complete the validation process.

## Methods

We developed a gamified assessment test relevant for appraising the cognitive abilities and personality characteristics of potential surgical residents and examined preliminary evidence for its validity, feasibility, and acceptability. In accordance with the contemporary understanding of validity as a unified concept, we collected and evaluated evidence related to 4 sources of validity: content, internal structure, response process, and relationships with other variables [27,28], although the evidence for relationships with other variables was limited and did not include test-criterion relationships. The evidence collected is based on both the procedures used in the development and revision of the test and the empirical data collected during the study. 

### Ethical Considerations

The study was approved by the ethics committee of the Hebrew University of Jerusalem (approval no. 13032023), and all participants provided informed consent. Participant data were stored using a unique fake identifier; the key linking these identifiers to real identities was kept in a password-protected file stored offline, ensuring that no identifying information was accessible online. Interns received US $75 for participating in the study, as well as feedback regarding their performance in both tests relative to the rest of the sample (the percentile rankings of their total scores). 

### Test Development

#### The GBA

The GBA used in this study was developed in cooperation with Benchmark.games LTD (Hungary), a company that produces GBAs for use in organizational hiring and recruitment. Tests are tailored to the organization’s needs, based on video games developed specifically for the assessment of various competencies (eg, analytical thinking, planning, or multitasking). Each test is administered on a standard computer and requires only a stable internet connection and a mouse.

**Figure 1.**
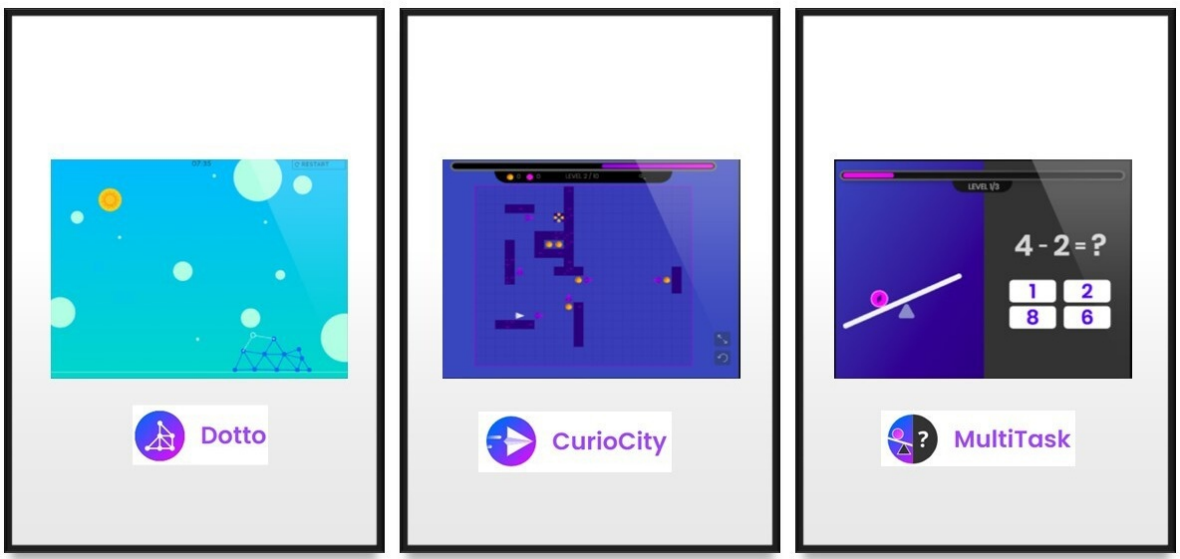
Illustrations of the video game assessments selected for the test. The games are shown in the order in which they appeared in the test.

The test developed for this study is based on three video games adapted to capture competencies needed by surgical residents: (1) Dotto, (2) CurioCity, and (3) MultiTask (refer to Figure 1). In the Dotto game, the goal is to build a structure by inserting and manipulating points and lines to reach a target while overcoming physics-based challenges. The game confronts examinees with a problem-solving situation that is not clearly defined, requiring them to discover the rules for solving the problem on their own. In CurioCity, examinees are tasked with finding their way through a maze to reach the target area. The game consists of 16 levels with varying requirements and levels of difficulty. Once again, some of the rules must be discovered by examinees, and some rules change as the game proceeds, to test the adaptability and flexibility of the examinees. Finally, in the MultiTask game, examinees are asked to perform 2 nonverbal tasks simultaneously (eg, a swing balancing task and a simple arithmetic task). The game has three levels, each using a different combination of 2 tasks. The initial versions of the games were developed by psychometricians and psychologists employed by Benchmark.games, and the games were validated using data from hundreds of employees by Benchmark.games for general personnel selection. For this study, all 3 games were modified based on feedback from the research team in 3 ways: levels that were insufficiently challenging for candidates with high abilities were excluded; tasks that assessed irrelevant competencies (eg, typing speed and accuracy) were replaced with tasks assessing competencies relevant for surgical trainees (eg, concentration and working memory); and, to ensure that the assessment would be objective and standardized, the instructions and demonstrations for each game were revised and improved. Instructions were provided in English and included both written instructions and video demonstrations. Furthermore, to ensure that the instructions were understood correctly, each game was preceded by a few minutes of practice. The initial version of the test was then pilot-tested with 8 medical students. Based on their feedback, changes were made in the instructions and in the test interface. The entire test takes about 45‐60 minutes to complete, with each game taking 15‐20 minutes.

The video games were selected to assess 14 relevant competencies: planning, problem-solving, ingenuity, goal orientation, self-reflection, endurance, analytical thinking, learning ability, flexibility, concentration, conformity, multitasking, working memory, and precision. Definitions of the competencies are provided in Table 1.

The competencies were drawn from a set of cognitive abilities and personality characteristics identified as relevant for selection of surgical residents in a previous phase of job analysis conducted by the research team [7]. However, the GBA does not assess some of the competencies which were identified as relevant to selection for surgical training (in particular, “soft skills” such as interpersonal skills, teamwork, leadership, and integrity). These competencies were not assessed in the present research because they are not susceptible to measurement using computerized and automated systems. The GBA was designed such that each game would elicit specific behaviors relevant to 2 or more of the 14 competencies, with each competency assessed using information obtained from one game (except for precision, which was assessed in all 3 games).

**Table 1.** Competencies assessed in the game-based assessment (GBA) test.

Competency	Description	Video game used to assess the competency
Planning	Ability to plan the steps required to solve the task, and to implement the plan in order to achieve the goal.	Dotto
Problem-solving	Ability to work through unexpected obstacles and challenges that arise during the task.	Dotto
Ingenuity	Ability to test the boundaries of a problem and to seek unique and creative solutions.	Dotto
Goal orientation	Ability to translate an intention into action (ie, to stay focused on achieving the goal).	Dotto
Self-reflection	Ability to learn from failure and to adopt a new approach.	Dotto
Endurance	Ability to invest effort for an extended period of time.	Dotto
Analytical thinking	Ability to collect, organize, and implement the information needed to solve the problem.	CurioCity
Learning ability	Ability to recognize “rules” quickly and effectively and apply them in the relevant situation.	CurioCity
Flexibility	Ability to adapt to changes in the situation.	CurioCity
Concentration	Ability to stay focused and to maintain high performance even in monotonous repetitive tasks.	CurioCity
Conformity	Ability and willingness to follow rules and instructions.	CurioCity
Multitasking	Ability to split attention between two tasks without harming performance.	MultiTask
Working memory	Ability to store and retrieve information in short-term memory.	MultiTask
Precision	Ability to perform the task in an accurate manner, with few errors.	All games

#### Scoring

The gamified tasks provide the stimuli by which the program measures candidates’ behavior. In each game, all actions of examinees (eg, mouse movements and key presses) are recorded and logged. Approximately 2000 data points are recorded for each 15-minute gameplay session. These raw data are then transformed into higher-level variables that describe a set of meaningful measurements (eg, time to first response, time between actions, accuracy, number of steps, and learning curve). Then, competency scores are calculated using an aggregation (ie, linear combination) of the relevant variables, with higher weight given to variables characterized by larger variance between candidates.

The initial mapping between different variables and competencies was determined by a team of psychologists and psychometricians employed by the company following a theory-driven approach [20]. This mapping was tested and improved based on empirical data from hundreds of employees, and variables that did not converge with the expected pattern were excluded from consideration. The mapping was then further validated based on correlations with other measures of cognitive abilities and personality (eg, Raven’s Progressive Matrices, the Stroop test, scales of the International Personality Item Pool, and the Bar-On Emotional Quotient Inventory; refer to Table S1 in Multimedia Appendix 1).

Competency scores are computed and standardized based on a norm created using a database of over 5000 observations. Scores are presented on a scale of 1‐10. For this study, we also calculated a total test score for each examinee by averaging the individual competency scores (with equal weight for each competency). To facilitate interpretation of the results, the total scores were then scaled to have a mean of 100 and a SD of 20.

### Validation

#### Sample and Procedure

To evaluate the test’s validity, feasibility, and acceptability, we recruited 30 experienced surgeons from 3 hospitals and 152 medical interns from 10 hospitals in Israel. The surgeons were asked to review the test and then complete a feedback questionnaire (see below). The interns were asked to complete the test, and their test data was collected and analyzed to evaluate the internal structure and psychometric characteristics of the test (discrimination, reliability, and correlations between competency scores). The interns also completed a feedback questionnaire similar to that filled in by the surgeons.

The expert surgeons were recruited using an email invitation. Email addresses of potential participants were obtained from hospital websites or from the Israeli medical association database. Recruitment continued until we had 30 participants. Surgeons who were willing to participate in the study were invited to review the gamified test and to complete the feedback questionnaire.

The interns were recruited using an invitation posted in relevant Facebook and WhatsApp groups. Recruitment continued until at least 150 participants were enrolled. Participants were invited to attend a session in which we administered the gamified assessment test and a separate technical aptitude test developed by Gazit et al [29]. The technical aptitude test included 10 basic tasks performed on the Lap-X VR laparoscopic simulator [30] and was designed to assess technical skills relevant for surgery such as dexterity, visuospatial perception, coordination, and arm-hand steadiness. The order of the tests varied, such that some participants started with the GBA and others with the technical aptitude test, with a short break between the two. The interns were told that each game in the GBA should take around 15‐20 minutes to complete. 

#### Questionnaire

The questionnaires filled in by the surgeons and interns were nearly identical. Participants in both samples were asked to provide four main ratings for each game: (1) its relevance for selecting candidates for surgical training (on a 5-point Likert scale, 1=not relevant, 5=extremely relevant); (2) its difficulty (also on a 5-point Likert scale, 1=very easy, 5=extremely difficult); (3) whether the time limit was sufficient (yes or no); and (4) whether the instructions were clear (yes or no). In addition, participants provided 2 ratings for the test as a whole: the relevance of the entire test and the comfort of the test platform (both on 5-point Likert scales, 1=not relevant or not comfortable, 5=extremely relevant or comfortable). Participants were also invited to share general comments and suggestions for improving each game and the whole test using free text. Finally, each participant provided demographic information (for interns: age, gender, dominant hand, desired training field [surgical or nonsurgical], and previous experience with video games; for the surgeons: age, gender, surgical specialty, and number of years working in the field). Previous experience with video games was reported on a 5-point scale (1=no experience, 5=very extensive experience).

#### Analyses

Some validity evidence is encompassed in the procedures used in the development of the test described above (selection of games and tasks based on job analysis; development of the games and scoring method by psychometricians and psychologists; and calculation of scores based on a norm sample). Further evidence of validity is derived from the empirical data collected in this study. In particular, internal structure evidence, response process evidence, and relationships with other variables were obtained from analysis of the interns’ test performance data. Content evidence, feasibility, and acceptability were obtained from the feedback questionnaires completed by both the interns and surgeons.

To analyze the performance data of the interns, we first examined the distribution of the competency scores and calculated Pearson correlations between them to support computation of a composite score for each participant, representing that participant’s total performance in the test (response process evidence of validity). We then conducted an item analysis to assess the discrimination of each competency and the reliability of the whole test, and a factor analysis to assess whether the structure of the test variables accords with what is theoretically expected (together these provide internal structure evidence for validity). Finally, we calculated correlations between participants’ scores in the gamified test and other variables: their demographic characteristics (age, gender, dominant hand, desired training field, and previous experience with video games) and their technical aptitude test scores (evidence of relationship to other variables).

To analyze the data from the feedback questionnaires of the interns and surgeons, we first calculated, for each sample, mean relevance and difficulty ratings for each game. We then analyzed the data on the time limits and clarity of instructions for each game, as described above, and calculated the mean relevance and comfort ratings for the whole test. Finally, we analyzed the general comments obtained from participants in the open-ended question to identify common remarks and suggestions. All statistical analyses were performed using R, version 4.2.2 (R Foundation for Statistical Computing, Vienna, Austria).

## Results

### Overview

In total, 152 interns (71 females, 46%) from 10 academic hospitals in Israel and 30 expert surgeons (4 females, 13%) from three academic hospitals in Israel participated in the study. Demographic characteristics of the participants are presented in Table 2.

**Table 2.** Demographic characteristics of study participants.

Group and characteristic	Values
Interns (n=152)	
Age in years, mean (SD)	28.3 (3.8)
Gender (female), n (%)	71 (46)
Dominant hand (left), n (%)	13 (9)
Desired training field, n (%)	
Surgical training	100 (65)
Nonsurgical training	36 (24)
Not decided	17 (11)
Experience with video games, n (%)	
No experience	22 (14)
Little experience	45 (29)
Moderate experience	46 (30)
Considerable experience	20 (13)
Very extensive experience	20 (13)
Expert surgeons (﻿n=30)	
Age in years, mean (SD)	53.8 (8.4)
Gender (female), n (%)	4 (13)
Years of experience, mean (SD)	13.5 (7.9)
Surgical specialty, n (%)	
General surgery	8 (27)
Gynecology	5 (17)
Orthopedics	10 (33)
Otorhinolaryngology–head and neck surgery	4 (13)
Urology	3 (10)

### Performance Data of Interns

**Figure 2.**
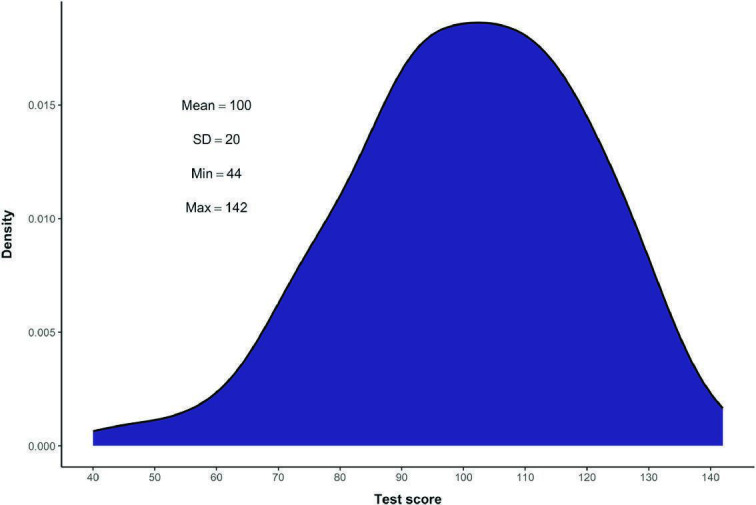
Distribution of total GBA test scores in the intern sample (n=152). GBA: game-based assessment.

First, competency scores and total test scores were calculated for each of the interns. The means and SDs of the competency scores and total scores are presented in Table 3. The total test scores ranged from 44 to 142 (a range of 98). Figure 2 displays the distribution of the total scores for the 152 interns (The distributions of the individual competency scores can be found in Figure S1 in Multimedia Appendix 1).

**Table 3.** Descriptive statistics and item analysis of the game-based assessment (GBA) test.

Competency	Mean	SD	Skew	Competency discrimination	Cronbach α if deleted
Planning	6.30	2.24	−0.29	0.64	0.72
Problem-solving	5.75	2.51	−0.08	0.47	0.73
Ingenuity	4.22	2.19	0.13	0.34	0.74
Goal orientation	4.79	2.38	−0.29	0.21	0.76
Self-reflection	4.87	3.09	0.14	0.23	0.76
Endurance	3.77	2.50	0.31	0.06	0.77
Analytical thinking	7.79	1.87	−1.13	0.46	0.74
Learning ability	7.23	1.97	−0.70	0.40	0.74
Flexibility	6.15	2.71	−0.25	0.36	0.74
Concentration	7.98	1.91	−1.30	0.34	0.75
Conformity	4.63	2.33	−0.10	0.17	0.76
Multitasking	7.39	2.38	−1.13	0.56	0.72
Working memory	6.22	3.47	−0.43	0.46	0.73
Precision	7.24	1.82	−0.71	0.71	0.72
Total test score^a^	100.00	20.00	−0.55	—^b^	—

a Cronbach α=0.76.

b Not applicable.

To support the calculation of a total test score based on the competency scores, we examined the Pearson correlations between the competency scores. Most of the correlations were high (refer to Table S2 in Multimedia Appendix 1). To support the internal structure of the test, an item analysis was then conducted to assess the discrimination of each competency and the reliability of the whole test (see Table 3).

The results showed good psychometric properties: the discrimination was satisfactory for all competencies (mean 0.39, SD 0.18), and the test’s internal reliability was high (α=0.76). In addition, we conducted exploratory factor analysis with Promax rotation on the 14 competencies. The Kaiser–Meyer–Olkin measure of sampling adequacy suggested that the data was factorable (Kaiser–Meyer–Olkin=0.69). The factor analysis yielded a 2-factor solution, such that seven competencies (analytical thinking, learning, flexibility, concentration, working memory, multitasking, and precision) loaded on one factor, and 6 competencies (planning, problem-solving, ingenuity, goal orientation, self-reflection, and endurance) loaded on the second factor. The only exception was conformity, which did not load on either of the factors. Based on our previous job analysis [7], we defined the first group as cognitive abilities and the second group as personality characteristics. The correlation between the two factors was 0.5. Detailed results for the factor loadings can be found in Table S3 in Multimedia Appendix 1.

We next calculated correlations between the total test scores in the GBA and external variables, including participants’ demographic characteristics and their scores in the separate technical aptitude test described earlier. No significant correlations were found between age, dominant hand, or desired training field and the total GBA scores. However, a significant difference emerged with respect to gender, such that males (mean 104.6, SD 16.8) scored significantly higher than females (mean 94.3, SD 21.9) on the gamified test (mean difference 10.9, 95% CI 3.1-17.6, *t*_150_=2.8, *P*=.002, Cohen *d*=0.52). This represents a small-to-medium effect size. In addition, we found a significant low positive correlation between the total GBA scores and reported amount of previous experience with video games (*r*_150_=0.26, *P*<.001). Interestingly, when we controlled for video game experience, the difference between the genders was no longer significant, suggesting that this difference is mainly due to different levels of video game experience.

Finally, we also calculated the correlation between the total GBA scores and scores in the technical aptitude test. We found a significant correlation between the 2 sets of scores (*r*_150_=0.46, *P*<.001). When controlling for video game experience, the correlation remained significant, though slightly reduced (semipartial *r*_152_=0.38, *P*<.001), suggesting that while gaming experience contributes to the association, the majority of the shared variance likely reflects underlying competencies relevant to both assessments. Supporting this interpretation, we found significant correlations between technical aptitude test scores and several nontechnical competencies measured by the GBA: planning, *r*_150_=0.28; problem-solving, *r*_150_=0.28; analytical thinking, *r*_150_=0.27; learning ability, *r*_150_=0.30; flexibility, *r*_150_=0.50; and precision, *r*_150_=0.30; all *P*<.001. In the absence of these 6 competencies, the total GBA scores showed no significant correlation with the technical aptitude test (*r*_150_=0.11, *P*=.17). These findings suggest that shared cognitive and behavioral attributes may play an important role in performance on both tests.

### Questionnaire Data

Table 4 presents the main results for the questionnaire data, including mean relevance and difficulty ratings for each game, and the rates at which participants judged the time limits as sufficient and the instructions as clear.

**Table 4.** Feedback of interns and expert surgeons on the relevance,^a^ difficulty,^b^ time limit,^c^ and clarity of instructions^d^ for each game in the game-based assessment (GBA) test.

Game and group		Relevance rating, mean (SD)	Difficulty rating, mean (SD)	Time limit, n (%)	Clarity of instructions, n (%)
Dotto					
	Interns	3.5 (0.8)	4.5 (0.4)	95 (62)	94 (61)
	Surgeons	3.8 (0.6)	4.2 (0.7)	22 (73)	21 (70)
CurioCity					
	Interns	3.8 (0.6)	2.9 (0.8)	151 (99)	144 (94)
	Surgeons	3.7 (0.7)	3.7 (0.6)	27 (90)	24 (80)
MultiTask					
	Interns	3.7 (0.6)	2.9 (0.7)	142 (93)	147 (96)
	Surgeons	3.6 (0.7)	2.5 (0.5)	29 (97)	27 (90)

a The relevance rating scale ranged from 1 to 5, with higher scores indicating greater relevance for selection of surgical residents (1=“not relevant”, 2=“slightly relevant”, 3=“moderately relevant”, 4=“very relevant”, 5=“extremely relevant”).

b The difficulty rating scale ranged from 1 to 5, with higher scores indicating greater difficulty (1=“very easy”, 2=“easy”, 3=“moderately difficult”, 4=“very difficult”, 5=“extremely difficult”).

c Participants were asked whether the time limit was sufficient for the task. The number in the table represents the number of interns and surgeons who responded “yes.”

d Participants were asked whether the instructions for the task were clear. The number in the table represents the number of interns and surgeons who responded “yes.” The instructions were modified slightly based on the surgeons’ feedback before the test was administered to the interns.

Addressing the latter first, overall, both the interns and expert surgeons regarded the time limits as sufficient (the lowest time limit approval rating was 62% of the interns for the Dotto game; for CurioCity and MultiTask, all ratings were 90% or above). Both samples also considered the instructions to be generally clear (again, the lowest approval rating was by the interns for the Dotto game, at 61%; see Table 4). Before the test was administered to the interns, some of the instructions were modified slightly and improved based on feedback provided by the expert surgeons either verbally or in writing.

The difficulty ratings varied between games, with the CurioCity and MultiTask games perceived overall as being moderately difficult, and the Dotto game largely perceived as very difficult to extremely difficult. The mean difficulty rating across the games and samples was 3.5 (SD 0.7), meaning that the test as a whole was perceived as moderately to very difficult. All games were considered by both the expert surgeons and the interns as relevant for assessing cognitive abilities and personality characteristics in the selection of candidates for surgical training (manifested in average ratings of 3.5 or above; see Table 4). The mean relevance rating across the games and samples was 3.6 (SD 0.1). Looking at the whole-test ratings, the mean relevance ratings were relatively high (interns: mean 3.6, SD 0.7; expert surgeons: mean 3.7, SD 0.6). In addition, the test platform was perceived as comfortable to use (interns: mean 4.2, SD 0.2; expert surgeons: mean 4.0, SD 0.3).

As noted, we also analyzed participants’ written feedback (in the free-text portion of the questionnaire), as well as feedback provided orally by the expert surgeons. Some of the surgeons indicated that their relevance ratings would have been higher if the tasks in the GBA were more directly related to surgical tasks and scenarios. Some participants also suggested that the test would be more relevant if it assessed other important competencies not covered in the current version, such as interpersonal skills, teamwork, leadership, and integrity. Finally, participants also expressed concern that prior experience with video games could affect performance on the test.

## Discussion

### Study Overview and Significance

This paper presents an innovative gamified test designed to assess cognitive abilities and personality characteristics relevant to the selection of surgical residents. While several studies have evaluated the use of GBAs in assessing applicants for employment, this is, to our knowledge, the first to evaluate their use in selecting surgical residents. As part of a broader program of validation research, this initial study provides preliminary evidence supporting the tool’s feasibility, acceptability, and validity.

### Evidence for Validity

#### Overview

On the basis of feedback from surgeons and interns regarding the test’s relevance, difficulty, and administration, the results of this study support the feasibility and acceptability of the test. We also present preliminary evidence concerning 4 of the 5 main components of construct validity: content, response process, internal structure, and relationships with other variables (the fifth component, consequences, could not be examined in this study) [27,28]. In some cases, the evidence is based on procedures used in the development and adaptation of the test; in others, it is based on empirical data collected during the study.

#### Content

In terms of content, the games used in the GBA were selected to assess relevant cognitive abilities and personality characteristics based on competencies identified in a previous job analysis [7]. The games were developed and validated by psychometricians and psychologists to evaluate these specific competencies, and both the interns and surgeons participating in the study rated the games as relevant for selecting candidates for surgical training. Some of the expert surgeons indicated that their relevance ratings would have been higher if the content of the games were more directly related to surgery or medicine. This weakens somewhat the content evidence for validity. However, the literature on gamification suggests that GBAs can effectively assess relevant competencies even when the game scenario seems unrelated to the profession [26]. Future studies should examine whether GBAs that more directly mimic job-related situations are more valid for selecting qualified candidates.

#### Response Process Evidence

Response process evidence of validity has 2 components. The first is the elimination of sources of error associated with test administration [28]. Toward this end, we provided detailed and thorough instructions for each game. The instructions were revised based on feedback provided by the expert surgeons before the test was administered to the interns. The ratings of both the expert surgeons and interns indicate that on the whole, the instructions were perceived as clear.

The second component of response process evidence is the appropriateness of the methods used to combine different performance parameters to produce a composite score. To support the calculation of a total test score based on the competency scores, we examined the correlations between the competency scores. Strong correlations were obtained, supporting the calculation of a composite performance score.

#### Internal Structure Evidence

Internal structure, as a source of validity, relates to the statistical or psychometric characteristics of the test. The item analysis conducted on the test data of the interns showed good psychometric properties, supporting the internal structure of the test. In addition, the factor analysis yielded two groups of competencies, one reflecting cognitive abilities and the other personality characteristics. This result is consistent with previous classifications of these competencies [4,7,31], and therefore also in keeping with the test’s expected internal structure.

#### Relationships With Other Variables

This source of evidence relates to the “degree to which these relationships are consistent with the construct underlying the proposed test score interpretation” [32]. Most commonly, this evidence is assessed based on correlations of assessment scores with a criterion measure of future workplace performance. While this type of evidence is indeed crucial for the validation of the current test, it was not available in this initial study.

Instead, the present analysis relies on a different methodology, namely, examining whether the relationships found in this study between test scores and external variables are consistent with what is known from the literature regarding the relationship between nontechnical competencies and those variables. Based on the data of interns, we calculated the correlations between participants’ performance on the gamified test and other variables.

As expected, no correlations were found with age, dominant hand, or the intern’s desired training field. We found relatively small but statistically significant correlations with both gender and self-reported video game experience, with males and frequent gamers obtaining higher GBA scores. Notably, the gender difference was largely accounted for by differences in video game experience, suggesting that the observed gender effect is explained by greater familiarity with video games among males. These findings are in line with other studies showing that gamers and males may potentially have advantages over nongamers and females in the context of GBAs [33,34], and they raise questions regarding the fairness of these tests. Since there is evidence that playing video games improves cognitive and mental abilities [35,36], it is unclear whether the correlation between video game experience and the gamified test scores found in this study reflects a genuine positive influence of video games on gamers’ abilities, or whether it is simply an artifact of the test format that may bias the selection process. Future research should examine whether changes in instructions, allowing more practice time before the test, or changes in GBA features and measures may eliminate these advantages [33]. In addition, further studies should examine whether increasing women’s exposure to video games in general would help to minimize this gender gap. However, it is important to note that the observed gender effect was small to medium in size, and the effect of video game experience was small. Thus, while caution is warranted, these differences should not be overstated. Until further evidence is available, the use of adjusted cutoffs or gender-specific norms may help avoid exacerbating the underrepresentation of women in surgical fields.

In addition, it is important to acknowledge that the GBA examined in this study does not encompass the full range of cognitive abilities and personality characteristics relevant for selecting surgical residents. Notably, key nontechnical competencies such as interpersonal skills, teamwork, leadership, and integrity were not addressed in the current assessment. Furthermore, the tasks included were primarily procedural and did not involve verbal abilities. As previous research has shown that males and females may excel in different domains—with females often demonstrating strengths in tasks that require verbal abilities [37] and interpersonal skills [38,39]—it is plausible that a more comprehensive assessment approach could mitigate the small gender differences observed in this study. For example, incorporating tools that evaluate verbal and interpersonal competencies might balance the overall selection outcomes. Future research should investigate whether expanding the assessment battery to include gamified situational judgment tests [21,40] or other instruments targeting these nontechnical domains could enhance fairness and reduce gender disparities in selection.

Moreover, we found a medium correlation between the gamified test scores and scores on a technical aptitude test performed using a virtual reality laparoscopic simulator. Since video game experience has been shown to correlate with initial performance on laparoscopic simulators [41], we considered the possibility that this shared factor may contribute to the observed association, that is, that previous video game experience might positively influence performance on both assessments. However, the correlation remained significant even after controlling for video game experience, suggesting that gaming experience only partially explains the relationship between the 2 tests.

In addition to this shared factor, our findings suggest that common underlying competencies may also play a role. Specifically, scores on the technical aptitude test were significantly associated with nontechnical competencies measured by the GBA, such as planning, problem-solving, analytical thinking, learning ability, flexibility, and precision. These results indicate that both assessments may tap into similar cognitive processes or behavioral tendencies. This interpretation is supported by prior research demonstrating meaningful correlations between nontechnical skills and performance on laparoscopic simulators [42-44].

To further disentangle the effects of gaming experience from shared competencies, future research should examine whether the correlation between GBA and laparoscopic simulator performance persists among individuals with previous laparoscopic experience. Alternatively, exploring the relationship between GBA scores and performance on open surgery tasks—which are not influenced by video game experience—could help clarify whether the observed correlation is driven by familiarity with gaming or by genuine overlap in nontechnical competencies.

Finally, as only 21% of the variance in GBA scores is explained by the technical aptitude test, it is clear that the GBA primarily measures competencies beyond those assessed by the laparoscopic simulator. This finding supports both the convergent and divergent validity of the GBA and aligns with its intended construct interpretation [32].

### Implications

Nontechnical skills are important for surgeons no less, and perhaps even more, than technical skills [7]. Indeed, many underlying causes of error within and outside the operating room originate from nontechnical aspects of performance [8]. Hence, training programs recognize the importance of assessing candidates’ cognitive abilities and personality characteristics when selecting each year’s cohort of surgical residents. Yet traditional assessment methods (academic achievement, curricula vitae, letters of recommendation, and interviews) are poorly correlated with later performance; and self-report measures, a potential alternative, are subject to bias and dishonesty.

The present study introduces an innovative solution for assessing relevant competencies: game-based assessment [21,22,25]. Building on existing GBAs developed for hiring and recruitment contexts, we implemented a systematic process to develop a gamified test tailored for surgical resident selection and conducted an initial investigation into its validity. Gamified assessment tests offer numerous advantages over other assessment approaches. First, they examine the entire solving process, as opposed to traditional tests which only examine the final product, allowing for a deeper understanding of the candidate’s competencies and work style. Compared to self-report measures, GBAs measure candidates’ actual behavior, which is harder to fake. Finally, gamified tests are based on automated scoring, thus minimizing the influence of bias in the selection process.

The present findings provide preliminary support for the feasibility, acceptability, and validity of the gamified test, suggesting that it may contribute to improving the selection of surgical residents by offering a potentially more reliable assessment of candidates’ abilities and attributes. It follows that implementing this test—or a similar tool—may assist program directors in identifying candidates with strong potential for success in surgical training. This improved selection process should, in turn, result in more capable surgical residents and surgeons, ultimately leading to better surgical outcomes and increased patient safety. Our findings may be relevant to nonsurgical training programs as well, since some of the competencies assessed in the gamified test developed in this study apply to residents in all medical fields.

The gamified test presented in this study does not assess all cognitive abilities and personality characteristics relevant for selecting surgical residents. As mentioned by the participants in this study, competencies missing in the present work include interpersonal skills, teamwork, leadership, and integrity. Future studies should examine whether other types of GBAs, such as gamified situational judgment tests [21], or other assessment methods may be useful in improving this area.

### Strengths and Limitations

This is the first study to examine the use of GBAs in selecting surgical residents, or indeed medical residents in any field. As such, one of its key strengths is use of a systematic process to develop a novel test for assessing candidates’ cognitive abilities and personality characteristics and to evaluate its validity, feasibility, and acceptability. Another strength is the large sample of expert surgeons (30) and interns (152) from various hospitals who provided data for statistical analysis (the interns) and feedback (both samples).

The study has some limitations. First, our participants came from a single country, thereby restricting the generalizability of our findings. However, it seems unlikely that the competencies we assessed are distributed differently among candidates from other nations. In addition, since the interns in our study were volunteers, it is possible that our sample does not represent the population of candidates for surgical training. Future studies should aim to recruit a more randomized and representative sample to ensure the findings are generalizable to the broader population of surgical trainees. However, the large variance in competency and test scores observed in our sample suggests that our sample was likely sufficiently representative of candidates with different qualifications. Finally, an important limitation of this study is the absence of evidence for test-criterion relationships. While we present data supporting various sources of validity, we have not yet assessed whether the GBA scores predict future performance in surgical residency. Given the high-stakes nature of surgical selection, establishing evidence for test-criterion relationships is critical before the tool can be adopted for widespread use. Longitudinal studies that track residents’ real-world performance over time are planned to address this essential aspect.

### Conclusions

The use of GBAs holds potential for contributing to improvements in resident selection. The present study presents an innovative gamified test designed to assess cognitive abilities and personality characteristics relevant to the selection of surgical residents. Preliminary evidence supports the feasibility, acceptability, and validity of the gamified test. However, further research is needed, particularly to assess evidence for test-criterion relationships, before the tool can be fully recommended for surgical resident selection.

## Supplementary material

10.2196/72264 Multimedia Appendix 1 Supplementary material.

## Abbreviations

GBA: game-based assessment
