# Supplementary material for: Game-Based Assessment of Cognitive Abilities and Personality Characteristics for Surgical Resident Selection: A Preliminary Validation Study
Source: JMIR Med Educ. 2025 Aug 15;11:e72264. doi: 10.2196/72264 (PMC12356604; doi:10.2196/72264)
Supplement: Multimedia Appendix 1 [file mededu-v11-e72264-s001.pdf]

Appendix

Table S1. Correlations Between Game-Based Assessment Competencies[a] and External Validation Measures (*N* = 402)

Figure S1. Distributions of Individual Competency Scores in the Intern Sample (*N* = 152)

Table S2. Correlations Between Competency Scores

Table S3. Factor Analysis of Competencies Assessed in the GBA Test

Table S1. Correlations Between Game-Based Assessment Competencies[a] and External Validation Measures ($N = 402$)

Competency	Validation measure	r (Pearson)[b]
Planning	IPIP – Playfulness scale	0.29
Problem-solving	Bar-On EQ test – Problem Solving subscale	0.35
Goal orientation	Bar-On EQ test – Independence subscale	0.44
Self-reflection	Bar-On EQ test – Reality Testing subscale	0.35
Endurance	Bar-On EQ test – Impulse Control subscale	0.43
	Bar-On EQ test – Stress Tolerance subscale	0.52
Analytical thinking	Raven's Progressive Matrices	0.29
Learning ability	Raven's Progressive Matrices	0.30
Flexibility	IPIP – Flexibility scale	0.29
	Stroop test	0.37
Concentration	Pieron test	0.44
Conformity	IPIP – Dutifulness scale	0.47
Precision	Pieron test	0.40

[a] Only competencies for which relevant, validated external measures were available during the prior mapping and validation phase are included in this table.
[b] All correlations were statistically significant ($p < .001$).

Abbreviations: IPIP: International Personality Item Pool; EQ: Emotional Quotient.

Figure S1. Distributions of Individual Competency Scores in the Intern Sample (N = 152)

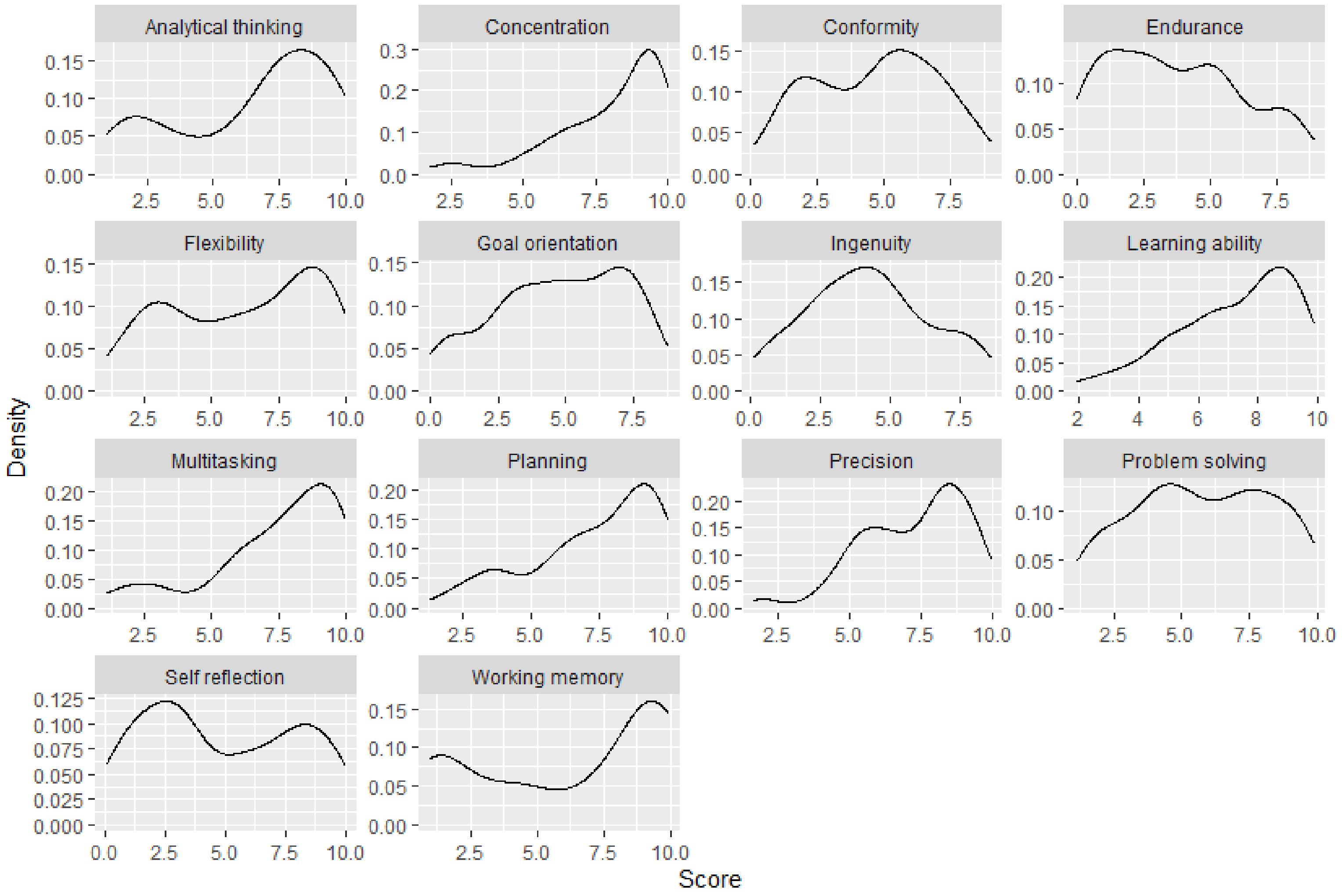

Table S2. Correlations Between Competency Scores

	PL	PS	I	G	S	E	A	L	F	CO	CF	M	W	PR
**PL**		0.57	0.33	0.46	0.21	0.14	0.46	0.25	0.13	0.24	0.08	0.36	0.39	0.09
**PS**	0.57		0.42	0.1	0.17	0.17	0.24	0.19	0.15	0.06	0.08	0.34	0.23	0.11
**I**	0.33	0.42		-0.12	0.17	-0.07	0.21	0.17	0.24	0.09	0.11	0.16	0.2	0.07
**G**	0.46	0.1	-0.12		0.11	0.12	0.12	-0.03	0.02	0.09	0.01	0.13	0.14	-0.08
**S**	0.21	0.17	0.17	0.11		0.27	0.07	0.09	0.05	0.03	-0.01	0.04	0.14	-0.08
**E**	0.14	0.17	-0.07	0.12	0.27		0.01	-0.07	-0.08	-0.11	0.05	-0.05	-0.05	-0.09
**A**	0.46	0.24	0.21	0.12	0.07	0.01		0.42	0.21	0.34	0.1	0.34	0.24	0.09
**L**	0.25	0.19	0.17	-0.03	0.09	-0.07	0.42		0.32	0.27	0.18	0.35	0.26	0.18
**F**	0.13	0.15	0.24	0.02	0.05	-0.08	0.21	0.32		0.28	0.16	0.36	0.23	0.1
**CO**	0.24	0.06	0.09	0.09	0.03	-0.11	0.34	0.27	0.28		0.08	0.27	0.2	0.01
**CF**	0.08	0.08	0.11	0.01	-0.01	0.05	0.1	0.18	0.16	0.08		0.11	0.06	0.12
**M**	0.36	0.34	0.16	0.13	0.04	-0.05	0.34	0.35	0.36	0.27	0.11		0.6	0.52
**W**	0.39	0.23	0.2	0.14	0.14	-0.05	0.24	0.26	0.23	0.2	0.06	0.6		0.21
**PR**	0.09	0.11	0.07	-0.08	-0.08	-0.09	0.09	0.18	0.1	0.01	0.12	0.52	0.21	

Abbreviations: PL – Planning, PS – Problem solving, I – Ingenuity, G – Goal orientation, S – Self-reflection, E – Endurance, A – Analytical thinking, L – Learning ability, F – Flexibility, CO – Concentration, CF – Conformity, M – Multitasking, W – Working memory, PR – Precision

Table S3. Factor Analysis of Competencies Assessed in the GBA Test

Competency	Factor 1	Factor 2
Planning		0.93
Problem-solving		0.52
Ingenuity		0.33
Goal orientation		0.43
Self-reflection		0.23
Endurance		0.22
Analytical thinking	0.82	
Learning ability	0.20	
Flexibility	0.40	
Concentration	0.42	
Conformity	-	-
Multitasking	0.84	
Working memory	0.45	
Precision	0.54	
Eigenvalues	3.54	1.66
Percentage of total variance	16	12

* Loadings => 0.2
